# Supplementary material for: Human movement and transmission dynamics early in Ebola outbreaks
Source: medRxiv. 2023 Dec 19:2023.12.18.23300175. Preprint. [Version 1] doi: 10.1101/2023.12.18.23300175 (PMC10775320; doi:10.1101/2023.12.18.23300175)
Supplement: 1 [file NIHPP2023.12.18.23300175V1-supplement-1.pdf]

medRxiv preprint doi: https://doi.org/10.1101/2023.12.18.23300175; this version posted December 19, 2023. The copyright holder for this preprint (which was not certified by peer review) is the author/funder, who has granted medRxiv a license to display the preprint in perpetuity.

It is made available under a CC-BY 4.0 International license .

Supplementary Information

Human movement and transmission dynamics early in Ebola outbreaks

Alexandria Gonzalez[1], Behnam Nikparvar[1], M. Jeremiah Matson[2], Stephanie N. Seifert[3], Heather D. Ross[4], Vincent Munster[5], Nita Bharti[1*]

1 Biology Department and Center for Infectious Disease Dynamics, Penn State University, University Park, PA, 16802, USA
2 University of Utah Health, Department of Internal Medicine, Salt Lake City, UT 84132, USA
3 Paul G. Allen School for Global Health, Washington State University, Pullman, WA, 99164 USA
4 Donald W. Hamer Center for Maps and Geospatial Information, Penn State University Libraries, Penn State University, University Park, PA, 16802, USA
5 Division of Intramural Research, Laboratory of Virology, National Institute of Allergy and Infectious Diseases, National Institutes of Health, Rocky Mountain Laboratories, Hamilton, Montana, 59840 USA

*Corresponding author: nita@psu.edu

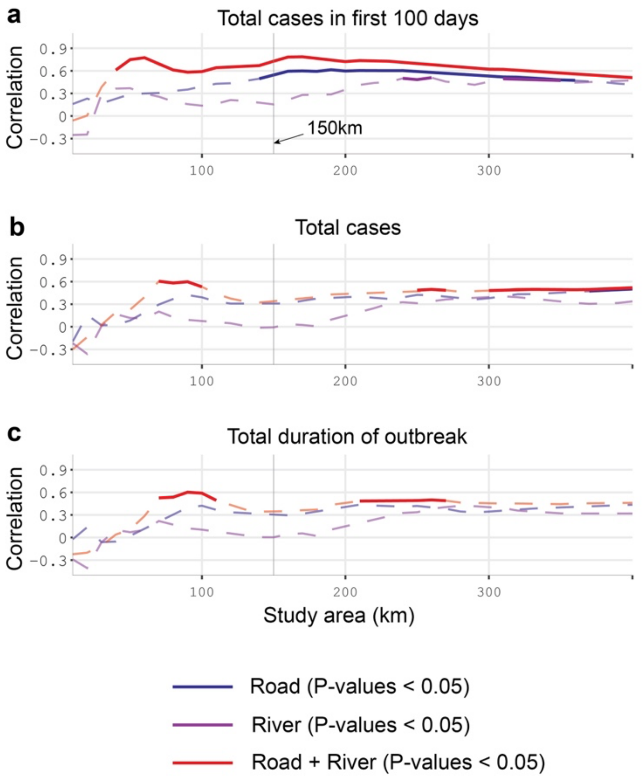

Figure S1. Sensitivity analysis of study area for correlations between transportation networks and outbreak measures: (A) cases in first 100 days, (B) total cases, and (C) total duration of outbreak. Dashed lines show p-values > 0.05; solid lines show p-values < 0.05; colors indicate transportation network.

medRxiv preprint doi: https://doi.org/10.1101/2023.12.18.23300175; this version posted December 19, 2023. The copyright holder for this preprint (which was not certified by peer review) is the author/funder, who has granted medRxiv a license to display the preprint in perpetuity.

It is made available under a CC-BY 4.0 International license .

Spillover location	Net change in road length (km)
Yambuku	+ 362.99
Tandala	+ 105.67
Mekouka	+ 9.48
Kikwit	+ 184.77
Mayibout	+ 0.77
Booue	+ 52.49
Mekambo/Mbomo	+ 57.48
Mbomo1	+ 13.78
Mbomo2	+ 0.93
Etoumbi	+ 14.42
Luebo07	+ 24.31
Luebo08	+ 15.59
Inkanamango	- 5.25
Likati	- 37.86
Bikoro	+ 162.38
North Kivu	- 119.55
Mbandaka	+ 13.83

Table S1. Net change in road length from 1963 to 2019 in the 150 km x 150 km study area surrounding the 17 spillover events that occurred in Central Africa. (+) Gain of road length, (-) loss of road length.

Outbreak measure

	**Cases in first 100 days**				**Total cases**				**Total duration (in days)**			
Study area	River	Road	Road & river	Inter-section	River	Road	Road & river	Inter-section	River	Road	Road & river	Inter-section
50 x 50	0.1365	0.0827	0.5580^{***S4A}	0.5589***	0.0137	0.0070	0.0519	0.0071	0.0050	0.0005	0.0114	0.0040
100 x 100	0.0201	0.1565	0.3540^{**S4B}	0.3940**	0.0065	0.1529	0.2812*	0.1367	0.0110	0.1780	0.3470^{*S2}	0.1143
150 x 150	0.0259	**0.3012***	**0.5377*****	0.4330^{**S3}	0.0000	0.0939	0.1146	0.0219	0.0000	0.0900	0.1150	0.0178
200 x 200	0.1276	0.3558**	0.5250^{***S4C}	0.4910**	0.0200	0.1531	0.1906	0.0735	0.0227	0.1717	0.2143	0.1091
300 x 300	0.2168	0.2694*	0.3890^{**S4D}	0.4306**	0.1626	0.1441	0.2311*	0.1494	0.1700	0.1184	0.2061	0.1970

Table S2. R-squared values for the transportation networks including total river length, total road length, total combined road and river length, and total intersections against outbreak measures: cases in first 100 days, total cases, and total duration in days for each study area examined (*p < 0.05, **p<0.01, ***p<0.001). All spillover events included. Upper case superscripts refer to figures that illustrate each relationship. Bold values correspond to Fig. 3A and Fig. 3B of the main paper, which illustrate these relationships.

Outbreak measure

	**Cases in first 100 days**				**Total cases**				**Total duration (in days)**			
Study area	River	Road	Road & river	Inter-section	River	Road	Road & river	Inter-section	River	Road	Road & river	Inter-section
50 x 50	0.1170	0.0738	0.5580***	**0.6020*****	**0.0370**	**0.0119**	**0.1352**	**0.2620***	**0.1190**	0.0003	**0.1630**	**0.1804**
100 x 100	0.0123	0.100	0.2710*	0.3160*	**0.0182**	0.0223	0.0010	0.0333	**0.1130**	0.0114	0.2670*	**0.1800**
150 x 150	**0.0267**	0.2450	0.4790**	0.4280**	**0.0690**	**0.1236**	0.0411	**0.0855**	**0.0915**	0.0538	**0.2460**	**0.1804**
200 x 200	0.1030	0.2890*	0.4560**	0.4590**	0.0002	**0.2890***	**0.2880***	**0.2330**	**0.2110**	0.1060	**0.2790***	**0.2060**
300 x 300	0.130	0.1970	0.2950*	0.3740*	0.0364	**0.2130**	**0.2420**	**0.2740***	**0.5710*****	0.0245	0.1960	0.1850

Table S3. R-squared values for the transportation networks including total river length, total road length, total combined road and river length, and total intersections against outbreak measures: cases in first 100 days, total cases, and total duration in days for each study area examined (*p < 0.05, **p<0.01, ***p<0.001). Outlier events

medRxiv preprint doi: https://doi.org/10.1101/2023.12.18.23300175; this version posted December 19, 2023. The copyright holder for this preprint (which was not certified by peer review) is the author/funder, who has granted medRxiv a license to display the preprint in perpetuity.

It is made available under a CC-BY 4.0 International license .

excluded. The outlier events are as follows: Meliandou Guinea in 2013 and North Kivu DRC in 2019. Upper case superscripts refer to figures that illustrate each relationship. Bold values are greater than when all spillover events are included.

	Outbreak measure											
	**Cases in first 100 days**				**Total cases**				**Total duration (in days)**			
Study area	River	Road	Road & river	Inter-section	River	Road	Road & river	Inter-section	River	Road	Road & river	Inter-section
50 x 50	**0.1780**	**0.1340**	**0.6140*****	**0.6110*****	**0.0180**	**0.0090**	**0.0520**	**0.0073**	**0.0070**	0.0004	0.0110	**0.0043**
100 x 100	**0.0613**	0.1560	**0.3800***	0.3720*	**0.0202**	**0.1620**	**0.2910***	0.1250	**0.0452**	0.1760	**0.3770***	0.0916
150 x 150	**0.0831**	**0.3160***	**0.5910****	0.4300*	**0.0020**	0.0920	**0.1180**	0.0162	**0.0111**	0.0760	**0.1260**	0.0085
200 x 200	**0.2030**	0.3540*	0.5200**	0.4650**	**0.0340**	0.1490	0.1820	0.0600	**0.0482**	0.1470	0.1920	0.0760
300 x 300	**0.2430**	**0.3260***	**0.4280***	**0.4490****	**0.1770**	**0.1620**	**0.2390**	0.1470	**0.1930**	**0.1400**	**0.2230**	0.1930

Table S4. R-squared values for the transportation networks including total river length, total road length, total combined road and river length, and total intersections against outbreak measures: cases in first 100 days, total cases, and total duration in days for each study area examined (*$p < 0.05$, **$p<0.01$, ***$p<0.001$). Subsequent events excluded. The subsequent events are as follows: Mayibout Gabon in February1996, Mbomo RC in November 2003, Etoumbi RC in May 2005, and Luebo DRC in September 2007. Upper case superscripts refer to figures that illustrate each relationship. Bold values are greater than when all spillover events are included.

	Outbreak measure											
	**Cases in first 100 days**				**Total cases**				**Total duration (in days)**			
Study area	River	Road	Road & river	Inter-section	River	Road	Road & river	Inter-section	River	Road	Road & river	Inter-section
50 x 50	**0.1490**	**0.1210**	**0.6070****	**0.6460****	**0.0514**	**0.0203**	**0.1550**	**0.3090**	**0.1800**	**0.0006**	**0.1920**	**0.2210**
100 x 100	**0.0418**	0.1070	0.3210	0.2970	0.0043	0.0124	0.0043	0.0145	**0.3470***	0.0033	**0.3860***	**0.1590**
150 x 150	**0.0782**	0.2620	**0.5390***	0.4200*	**0.0334**	**0.1150**	0.0568	**0.0607**	**0.2980**	0.0399	**0.3360***	**0.1640**
200 x 200	**0.1650**	0.2940	0.4570*	0.4370*	0.0050	**0.2800**	**0.2800**	**0.1870**	**0.4380***	0.0776	**0.2820**	**0.1620**
300 x 300	0.1510	0.2580	0.3450*	0.4050*	0.0484	**0.3150**	**0.3220**	**0.3100**	**0.7930*****	0.0460	**0.2740**	**0.2150**

Table S5. R-squared values for the transportation networks including total river length, total road length, total combined road and river length, and total intersections against outbreak measures: cases in first 100 days, total cases, and total duration in days for each study area examined (*$p < 0.05$, **$p<0.01$, ***$p<0.001$). Subsequent and outlier events excluded. The subsequent events are as follows: Mayibout Gabon in February1996, Mbomo RC in November 2003, Etoumbi RC in May 2005, and Luebo DRC in September 2007. The outlier events are as follows: Meliandou Guinea in 2013 and North Kivu DRC in 2019. Upper case superscripts refer to figures that illustrate each relationship. Bold values are greater than when all spillover events are included.

medRxiv preprint doi: https://doi.org/10.1101/2023.12.18.23300175; this version posted December 19, 2023. The copyright holder for this preprint (which was not certified by peer review) is the author/funder, who has granted medRxiv a license to display the preprint in perpetuity.

It is made available under a CC-BY 4.0 International license .

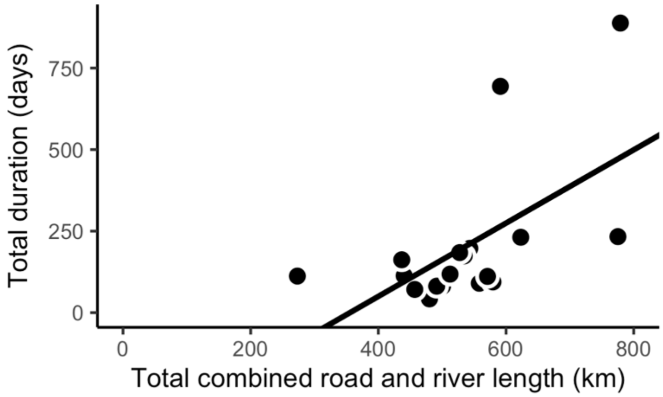

Figure S2. Linear Regression model of the total duration in days of each outbreak and the total combined road and river length in km in each 100x100 study area surrounding the spillovers (R-squared = 0.3470 , p = 0.0101).

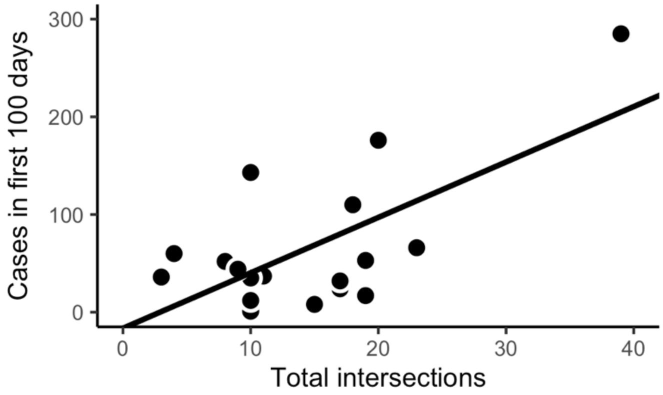

Figure S3. Linear Regression model of the total cases in the first 100 days of each outbreak and the total number of intersections in a 150 km x 150 km study area surrounding the spillovers (R-squared = 0.4338, p = 0.0030).

medRxiv preprint doi: https://doi.org/10.1101/2023.12.18.23300175; this version posted December 19, 2023. The copyright holder for this preprint (which was not certified by peer review) is the author/funder, who has granted medRxiv a license to display the preprint in perpetuity.

It is made available under a CC-BY 4.0 International license .

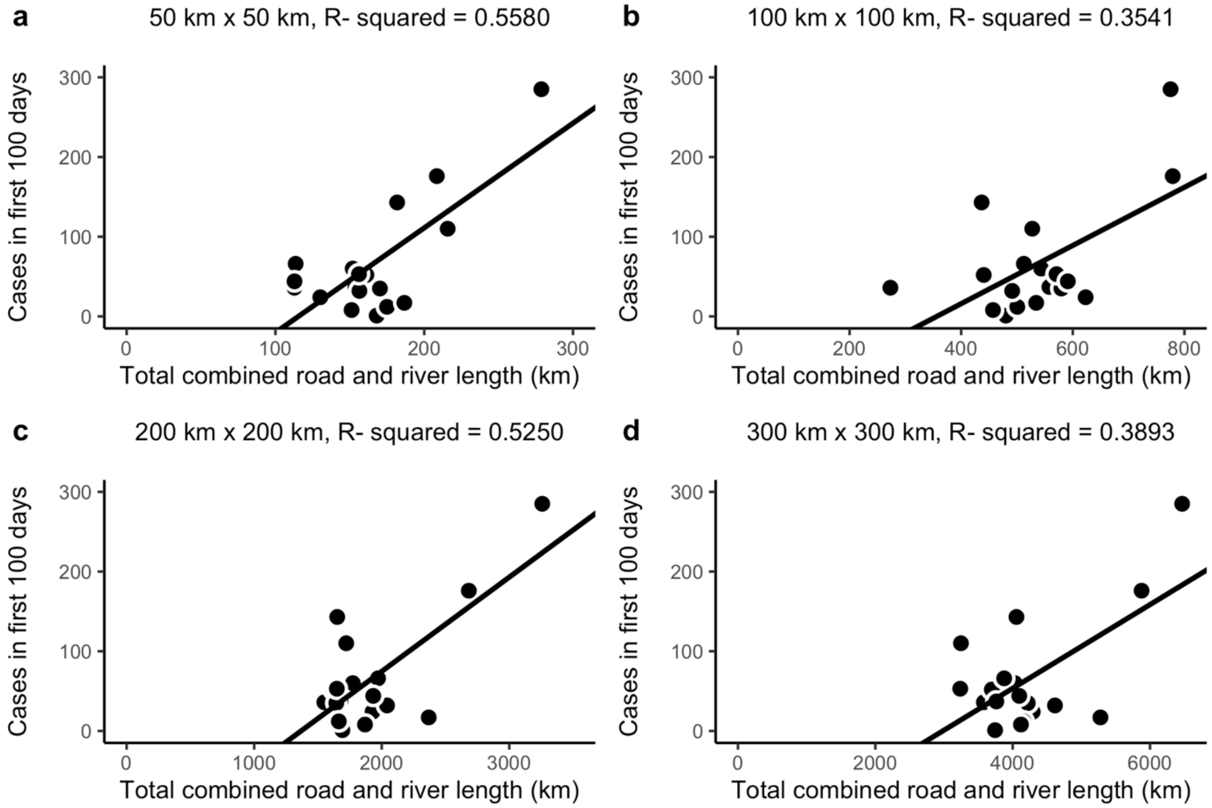

Figure S4. Linear Regression models of the total cases in the first 100 days and the total combined road and river length in km in a **(a)** 50 km x 50 km study area (R- squared = 0.5580 , p = 0.0004), **(b)** 100 km x 100 km study area (R-squared = 0.3541 , p = 0.0092), **(c)** 200 km x 200 km study area (R-squared = 0.5250 , p = 0.0007), **(d)** 300 km x 300 km study area (R-squared = 0.3893 , p = 0.0057).
